# Molecular Mechanisms and Genome-Wide Aspects of PPAR Subtype Specific Transactivation

**DOI:** 10.1155/2010/169506

**Published:** 2010-08-31

**Authors:** Anne Bugge, Susanne Mandrup

**Affiliations:** Department of Biochemistry and Molecular Biology, University of Southern Denmark, 5230 Odense, Denmark

## Abstract

The peroxisome proliferator-activated receptors (PPARs) are central regulators of fat metabolism, energy homeostasis, proliferation, and inflammation. The three PPAR subtypes, PPAR*α*, *β*/*δ*, and *γ* activate overlapping but also very different target gene programs. This review summarizes the insights into PPAR subtype-specific transactivation provided by genome-wide studies and discusses the recent advances in the understanding of the molecular mechanisms underlying PPAR subtype specificity with special focus on the regulatory role of AF-1.

## 1. Introduction

The peroxisome proliferator-activated receptors (PPARs) constitute a subgroup of the nuclear receptor (NR) family. The founding member of the family, PPAR*α*, was identified in 1990 and named by its ability to become activated by chemicals known to induce peroxisome proliferation in rodents [1]. Subsequently, the two other PPAR subtypes, PPAR*β*/*δ* and PPAR*γ*, were identified by homology screens [2, 3]. The three PPAR subtypes are encoded by distinct genes located on different chromosomes (reviewed by [4]). Alternate promoter usage and splicing give rise to two different protein isoforms from the PPAR*γ* gene called PPAR*γ*1 and PPAR*γ*2, with the latter containing 30 additional amino acids at the N-terminus (Swiss-Prot http://www.expasy.org/). All three PPAR subtypes can be activated by a large variety of fatty acids and fatty acid metabolites, such as hydroxylated eicosanoids, prostaglandins, and leukotrienes, and by many synthetic compounds. PPAR*α* is specifically activated by fibrates and other hypolipidemic drugs, whereas PPAR*γ* is activated by the insulin-sensitizing, antidiabetic thiazolidinedione drugs [4].

The PPARs play important regulatory roles in numerous cellular processes related to metabolism, inflammation, differentiation, proliferation, and atherosclerosis (reviewed by [5, 6]). The three subtypes display dissimilar patterns of tissue distribution and activate both overlapping and distinct sets of target genes. Most notably, whereas PPAR*α* [7, 8] and -*β*/*δ* [9] are potent activators of genes involved in lipid oxidation, PPAR*γ* stands out by its additional ability to activate lipogenic genes and adipocyte differentiation [10, 11]. In fact, PPAR*γ* is obligate for adipocyte differentiation and is sufficient to transform many nonadipogenic cell lines into adipocyte-like cells [12, 13]. As a reflection of these subtype-specific properties, PPAR*α* and PPAR*β*/*δ* are highly expressed in tissues with high *β*-oxidation rates such as liver, muscle, heart, and brown adipose tissue. By contrast PPAR*γ* is highly expressed in adipose tissue, and PPAR*γ*2 is fat selective, whereas the PPAR*γ*1 isoform is expressed at low levels in several tissues, including colon, spleen, liver, and muscles. PPAR*β*/*δ* is the most ubiquitously expressed subtype with the highest levels found in the intestines and keratinocytes (see [14] and reviewed by [5]). 

Like most NRs, the PPAR protein structure consist of four domains: The N-terminal A/B-domain containing the ligand-independent activation function 1 (AF-1), the C-domain, which is the DNA-binding domain (DBD), the D-domain, also called the hinge region, and finally the E-domain, commonly referred to as the ligand binding domain (LBD). The E-domain contains the ligand-dependent AF-2, which is highly dependent on the C-terminal helix 12. While the A/B -and D-domains are only poorly conserved between the PPAR subtypes, the C -and E-domains share a high degree of sequence and structural homology (reviewed by [4]). In fact, the C-domains are completely interchangeable between the PPAR subtypes and appear to have no effect on specificity [15, 16]. The PPARs bind DNA as obligate heterodimers with members of the retinoid X receptor (RXR) family of nuclear receptors to modified direct repeat 1 elements (DR1) with the consensus sequence 5′-AACTAGGNCA A AGGTCA-3′. The PPARs occupy the 5′ extended half site of the binding site [17].

Given the important role of the PPARs in regulation of metabolism, inflammation, differentiation, and cellular growth, a large number of specific and potent synthetic ligands have been generated. This has spurred a huge interest in understanding the molecular mechanisms of PPAR transactivation and in genome-wide approaches to identify new PPAR target genes. These studies have provided the field with important insights into how different ligands modulate the transactivation capacity of the PPARs and to what extent the individual PPAR domains are involved in ensuring subtype-specificity by enabling or preventing transactivation of specific subsets of target genes. This paper focuses on the recent advances in understanding PPAR subtype-specific transactivation as seen from a molecular and a genome-wide perspective. In particular, the regulatory role of the AF-1 in maintaining PPAR subtype-specificity and transactivation capacity is reviewed. 

## 2. Molecular Mechanisms of PPAR Subtype Specific Transactivation

The ability of the individual PPAR subtypes to induce very different cellular fates is intriguing because they share a high degree of sequence- and structural homology and activate overlapping sets of target genes. Nevertheless, the PPARs maintain a high degree of subtype-specificity when expressed in a given cell line at comparable levels [11, 18, 19] and adenoviral expression of PPAR*γ*1 in mouse liver leads to induction of several genes which are not readily activated by PPAR*α*, that is, genes involved in lipid accumulation and adipogenesis [20]. These results indicate that although the chromatin setting ultimately determines the accessibility of the PPAR response elements, intrinsic properties of the individual PPAR subtypes are key determinants of the gene programs that can be activated. Mechanisms maintaining subtype-specificity are of significant general interest because subtype selective gene regulation is a recurrent theme among transcription factor families, and therefore several attempts have been made to address this issue. These studies reported that the PPAR subtypes differ only little in their ability to transactivate artificial promoter reporter constructs in transient transfections [21–24] and display limited specificity in their binding to naked DNA containing target gene PPAR response elements (PPREs) in mobility shift assays, although poorly conserved PPREs preferentially bind PPAR*γ*:RXR heterodimers [25]. By contrast, work from our laboratory has shown that in the endogenous chromatin setting, the binding level of a particular PPAR subtype to a given genomic PPRE generally correlates with its potential to transactivate the corresponding target gene, although exceptions clearly exist [11].

### 2.1. The LBD and AF-2

Because of the obvious therapeutic potential for modulating PPAR transactivation through the administration of ligands that bind to the E-domain, the cofactor interaction surfaces and the molecular mechanisms underlying activation of the AF-2 have been extensively studied. The recent publication of an almost complete structure of the DNA bound PPAR*γ*:RXR*α* heterodimer [26] represents a major breakthrough in the understanding of the positioning of the PPAR*γ* and RXR domains relative to each other and their interactions. Unfortunately, the PPAR*γ* and RXR*α* A/B-domains could not be crystallized, most likely due to their high mobility and lack of internal structure. However, the overall impression from this study is that the PPAR*γ* LBD is the centerpiece of the complex, around which all other domains from both PPAR*γ* and RXR*α* are arranged [26]. This accentuates previous descriptions of extensive interdomain cross-talk in the PPARs [27, 28]. 

The PPAR*γ* LBD, is composed of 13 *α*-helices and a small four-stranded *β*-sheet that forms a large (approx. 1300 Å^3^) T-shaped hydrophobic ligand-binding pocket typical for the promiscuous NRs, such as PPARs and the pregnane X receptor (PXR), that bind many different ligands with low affinity [29]. The ligand-binding pocket of PPAR*β*/*δ* is narrower than those of PPAR*γ* and PPAR*α*, which appears to be a major determinant of ligand binding, as it prohibits binding of TZDs and severely decreases the affinity of PPAR*β*/*δ* towards fibrate ligands due to the bulky acid and alkyl groups on these compounds [30]. PPAR*α* contains the most lipophilic ligand binding pocket, which potentially explains why PPAR*α*, as opposed to PPAR*β*/*δ* and PPAR*γ*, binds a variety of saturated fatty acids [31]. An additional layer of ligand binding specificity is imposed by the size and charge of the amino acids lining the ligand binding pocket [30]. Ligand-binding affects the stability of the PPARs with liganded PPAR*α* being transiently stabilized [32] while proteasomal degradation of PPAR*γ* is increased upon ligand binding [33].

The structural basis for AF-2-mediated transcriptional activity is a ligand-induced conformational change in the LBD, causing the most C-terminal helix 12 to fold up against the core [30]; thereby generating an activation surface, often described as a charge clamp, onto which coactivators can dock. Many coactivators bind to the NR E-domains through a motif with the consensus sequence LXXLL, which facilitates direct interaction with the charge clamp. In contrast, corepressors often have a LXXXIXXX(I/L) motif that interacts with an overlapping surface but is unable to fit into the charge clamp (reviewed by [34]). The variations in the primary sequences of the PPAR LBDs results in slight differences in the cofactor interaction surfaces and ligand-induced conformations of these domains [35, 36] and PPAR subtype-specificity is thought to be partly imposed by differential affinity of the receptors towards the individual cofactors [37, 38]. Thus, the cofactor expression pattern in a specific cellular context may favor transcriptional activation of one PPAR subtype over the others. 

Probably, the transcriptional activity of the PPARs should not be regarded as determined by a static positioning of helix 12 in either the “on” or “off” position. Rather, it appears that ligand-binding shifts the equilibrium of the different helix 12 positions in the receptor population towards the more active conformations (reviewed by [39]) [40]. Helix 12 is absolutely necessary for the activity of AF-2, and PPAR mutants with helix 12 partly or completely deleted are dominant negative inhibitors of PPAR signaling [41, 42]. Interestingly, the requirement for helix 12 does not appear to equalize a requirement for ligand-binding. Recently, it has been demonstrated that a natural PPAR*γ* mutant that is impervious to activation by virtually all known agonists has intact adipogenic potential [43]. Indeed, the PPARs display high basal transcriptional activity that can be explained by the AF-1 in the A/B-domain and the presence of endogenous ligands. In addition, ligand-independent recruitment of coactivators to the AF-2 has been observed in overexpression and *in vitro* studies, indicating that in addition to the AF-1, the AF-2 also contributes to ligand-independent transactivation. Perhaps, this is possible because the shift in the positioning of helix 12 upon ligand-binding is not as pronounced for the PPARs as for the steroid NRs [44, 45].

### 2.2. The Elusive Structure of the PPAR A/B-Domain

Compared to the AF-2, the mechanisms for AF-1-mediated transcriptional activity are less well-understood despite several publications pointing to an important role for the A/B-domains in maintaining PPAR subtype-specificity [15, 16, 46]. The fact that it so far has proven impossible to crystallize a NR A/B-domain indicates that these domains are poorly structured, a notion that has been confirmed by experiments using deuterium exchange mass spectrometry [26], circular dichroism spectroscopy, nuclear magnetic resonance spectroscopy [47, 48], or limited proteolysis [49, 50]. It has been suggested that secondary structure formation is an important step in AF-1-mediated transactivation and both the PPAR*α* [51] and glucocorticoid receptor (GR) [47] AF-1 display *α*-helical characteristics in the presence of trifluoroethanol, a strong *α*-helix stabilizing agent. Furthermore, mutational analyses of the PPAR*α* [51], GR [52, 53], and hepatocyte nuclear factor 4 (HNF-4) [54] AF-1 domains have shown that conservation of the hydrophobic amino acids, that potentially could be involved in *α*-helix formation is especially important for the transcriptional activity, while mutation of individual acidic amino acids made less of an impact, suggesting that *α*-helix formation is an important step in AF-1-mediated gene activation. Interestingly, it was recently shown that mitogen-activated protein kinase (MAPK) phosphorylation of serine 211 in the glucocorticoid receptor A/B-domain induces formation of secondary or tertiary structure in this region which facilitates interaction between the AF-1 and coregulators thereby leading to enhanced transcriptional activity [55]. Another model proposes that, as individual coactivators offer different surfaces for unstructured activation-domains to fold up on, distinct conformations could be induced by the different coactivators, thereby resulting in differential transcriptional activity or specificity [56]. Several of the cofactors reported to interact with NR A/B-domains have no sequence or known structural homology, and this model offers an attractive explanation for how that is possible. 

### 2.3. PPAR Transcriptional Activity Is Regulated by Modification of the A/B-Domain

It is well-established that posttranslational modifications of the PPAR A/B-domains influence the transcriptional activity of both the AF-1 and AF-2 through various mechanisms. The PPAR*α* and –*γ* A/B-domains, but not the PPAR*β*/*δ* A/B-domain, are modified by phosphorylation. MAPK- phosphorylation of serine 12 and 21 in the PPAR*α* A/B-domain, enhances the transcriptional activity by transiently increasing receptor stability through reduced ubiquitination [32]. Oppositely, phosphorylation of serine 76 by glycogen synthase kinase 3 (GSK3) leads to increased ubiquitination and degradation of PPAR*α* [93]. MAPK mediated phosphorylation of serine 82 in the PPAR*γ*1 A/B-domain (corresponding to serine 112 in PPAR*γ*2) has been shown to inhibit both ligand-dependent and independent transactivation [94], the former by decreasing the ligand-binding affinity of the receptor [27]. Interestingly, it was recently published that the phosphorylated PPAR*γ* AF-1 domain is bound by the peptidyl-prolyl isomerase Pin1, whereby both polyubiquitination and the transcriptional activity of PPAR*γ* is inhibited, possibly due to the decreased turnover rate of the receptor [95]. Oppositely, it has also been reported that S112 phosphorylation of PPAR*γ*2 by a constitutively active MAPK kinase (MEK) [96] or by cyclin-dependent kinase 9 (Cdk 9) residing in the positive transcription elongation factor b complex (P-TEFb) results in increased transcriptional activity [71]. Thus, it appears that the cellular and/or molecular context determines the transcriptional effect of PPAR*γ* A/B-domain phosphorylation. Mice homozygous for the S112A mutation are indistinguishable from the wild types on a normal diet, but they are significantly more glucose tolerant in the setting of diet-induced obesity [97], an effect analogous to the outcome of PPAR*γ* activation by TZD treatment. In line with phosphorylation of S112 decreasing the insulin sensitizing actions of PPAR*γ*, humans carrying a P115Q mutation, that blocks phosphorylation of serine 114 (corresponding to serine 112 in mice), are extremely obese but are also less insulin-resistant than expected based on their degree of obesity [98]. In addition to affecting the activity of PPAR*γ* through regulation of MAPK, MEK has also been reported to interact directly with PPAR*γ* and promote its nuclear export [99]. Recently, it was furthermore reported that phosphorylation of serine 16 and 21 of PPAR*γ* by Casein-kinase-II likewise promotes shuttling of PPAR*γ* from the nucleus to the cytosol [100]. Besides phosphorylation, PPAR*γ* transactivation is also repressed by conjugation of small ubiquitin-related modifier (SUMO) to lysine 107 in the A/B-domain [101].

### 2.4. The PPAR A/B-Domain Is Involved in the Recruitment of Cofactors

In addition to regulating the PPAR transcriptional activity by affecting receptor stability, cellular compartmentalization, and perhaps interdomain communication in response to the posttranslational modification status, the PPAR A/B-domains are involved in recruiting a handful of cofactors. The PPAR*γ* AF-1 is the most well-described of the three PPAR A/B-domains and the coactivators Tat-interacting protein 60 (Tip60) [82] and PPAR*γ* coactivator-2 (PGC-2) [15] but also the corepressor *tribbles* homolog 3 (TRB3) [92] are recruited to PPAR*γ* exclusively through binding to the A/B-domain. Both PPAR*α* and PPAR*γ* have been shown to bind the histone acetyl transferase (HAT) coactivators p300 and CREBP-binding protein (CBP) through interaction surfaces in both the A/B-and E-domains in GST-pulldown studies [61]. The significance of the A/B-domain interaction was previously unknown, but we have recently shown that recruitment of p300 and CBP is compromised by deletion of the PPAR*γ* A/B-domain specifically on the PPREs of the target genes that required AF-1 activity to become fully activated [46]. The SWI/SNF chromatin remodeling complex BRG1-associated factor 60c (BAF60c) subunit which interacts directly with PPAR*γ* likewise appear to have the potential to bind both the A/B- and E-domains, but the AF-1 interaction is stronger and ligand-independent [58]. PPAR*α* has been shown to be coactivated by binding of the target gene product bifunctional enzyme (BFE) to the A/B-domain [57], and the ubiquitin ligase murine double minute 2 (MDM2) is bound by the N-terminal of all three PPAR subtypes [69]. In addition, we have recently demonstrated that the Mediator complex is tethered to the PPAR*γ* A/B-domain through the MED14 subunit and that MED14 is required for full transcriptional activation of PPAR*γ* subtype-specific genes by PPAR*γ* [102]. A complete list of the cofactors currently known to interact with the PPARs is shown in Table 1.

### 2.5. The A/B-Domains Play an Important Role in Maintaining PPAR Subtype-Specificity

Because the A/B-domain is the least conserved region among the PPARs, it has long been suspected that subtype-specificity, and target gene selectivity is completely or partly mediated through this domain. This hypothesis has been investigated by deleting the A/B-domain or by constructing chimeric PPARs where domains have been swapped between the subtypes.

Despite the undisputed observation that the PPAR*α* and -*γ* A/B-domains are potent transactivators when expressed as GAL4-fusion proteins [51] there has been controversy regarding the physiological importance of the activity of the PPAR A/B-domains. Deletion of the A/B-domain was reported to have no effect [82, 103] or to significantly decrease PPAR-mediated expression from a reporter plasmid in transient transfections [51]. Interestingly, deletion of the A/B-domain affected the transcriptional activity of PPAR*α* differentially depending on the target gene promoter used in the reporter construct. One study employed the acyl-CoA oxidase promoter and found that the A/B-domain contributed significantly to the transcriptional activity of PPAR*α* [51], while another study showed that luciferase expression driven by the cytochrome P450 4A6 promoter was completely unaffected by deletion of the AF-1 [103]. We have recently on a global scale shown that deletion of the PPAR*γ* A/B-domain selectively decreases the transactivation potential of PPAR*γ* on the highly subtype-specific target genes. We found that out of 257 PPAR*γ*-induced genes only 25 are dependent on the PPAR*γ* A/B-domain to become fully activated in the presence of the TZD rosiglitazone. The A/B domain dependent genes are the highly PPAR*γ* selective target genes many of which are involved in lipid storage. Notably, in the absence of synthetic agonist, transactivation of this subgroup of genes in particular relies almost exclusively on the PPAR*γ* A/B-domain [46].

**Figure 1:**
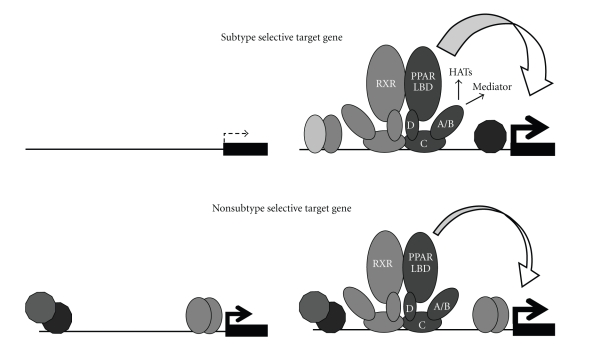
**The PPAR A/B-domains potentiate transactivation of the highly subtype selective target genes**. Illustration of the differential requirement for the PPAR A/B-domains in the transactivation of highly subtype selective and non-subtype selective target genes. The PPAR subtype specific target genes are generally expressed at very low levels in the absence of PPARs, but expression is dramatically increased upon introduction of full length exogenous PPAR. The activities of the PPAR A/B-domains are necessary to obtain this potent induction of the highly subtype specific target genes, presumably by facilitating recruitment and tethering of histone acetylase complexes (HATs) and the Mediator complex. Conversely, non-subtype selective PPAR target genes are usually already expressed at high levels in the absence of PPARs, and their expression levels are only increased by a few fold in response to ectopic PPAR expression. The PPAR A/B-domains appear to be dispensable for transactivation of this group of target genes.

The importance of the PPAR A/B-domains in maintaining subtype-specificity has been indicated by several studies showing that these domains are not interchangeable. Thus, although both the *α* and *γ* A/B-domains contain potent activation functions [51], they cannot functionally substitute for each other as evidenced by the observation that a chimera consisting of the PPAR*α* A/B-domain and the PPAR*γ* CDE-domains is able to transactivate the PPAR*γ* selective target genes similar to that of PPAR*γ*CDE [46]. However, the A/B-domains of PPAR*γ* and PPAR*α* can impose a partial subtype-specific activation in the context of a noncognate receptor. Spiegelman and coworkers showed that swapping the PPAR*γ* A/B-domain on to the nonadipogenic PPAR*β*/*δ*CDE conferred adipogenic potential to this receptor subtype [15]. Subsequently, Tontonoz and coworkers reported that the PPAR A/B-domains function to restrict target gene activation in the context of the cognate receptor and showed that A/B-domain deleted PPAR*β*/*δ* (PPAR*β*/*δ*CDE) can induce adipogenesis. This study thereby raised the question as to what degree the adipogenic potential of the PPAR*γ* A/B-domain-PPAR*β*/*δ*CDE chimera used in the Spiegelman study arose by the addition of the PPAR*γ* A/B-domain or the lack of the PPAR*β*/*δ* A/B-domain [13, 16]. In agreement with the A/B-domains conferring subtype-specificity to the PPARs in part by limiting nonselective target gene activation, we have shown that compared to the full length receptors, the A/B-domain deleted PPAR*α*CDE and -*γ*CDE are far better transactivators of the noncognate highly subtype selective PPAR target genes normally activated by the opposite subtype. However, reminiscent of the Spiegelman data we also found that addition of the PPAR*α* A/B-domain greatly enhances the ability of PPAR*γ*CDE to activate a PPAR*α* specific target gene [46]. Thus, it appears that the A/B-domains contribute to maintaining PPAR subtype-specificity by both potentiating activation of the highly subtype selective target genes, and by restricting nonselective target gene activation exclusively in the context of the cognate CDE domains. By contrast, the A/B-domain plays only a minor role in the activation of the target genes shared between the subtypes (Figure 1).

## 3. Genome-Wide Approaches to Mapping PPAR Subtype-Specific Transactivation

The PPAR transcriptome in cells and tissues has been mapped by ectopically expressing a particular subtype and/or treating with a specific agonist and mapping the changes in gene expression using array technology. More recently the combination of chromatin immunoprecipitation (ChIP) with microarray analysis (ChIP-chip), high throughput sequencing (ChIP-seq), or with pair end-tagging sequencing (ChIP-PET) has allowed the mapping of the PPAR cistrome in cells and tissues following various treatments. These global techniques have led to important insights into the role of the different PPAR subtypes in the regulation of metabolism and differentiation and into the action of PPAR agonists.

### 3.1. Expression Array Studies

Although microarray studies of NIH-3T3 fibroblast overexpressing PPAR*β*/*δ* have confirmed that this PPAR subtype rightfully is recognized as being an inducer of genes involved in energy expenditure and *β*-oxidation of fatty acids [16] it appears that at least in insulin-resistant (*db/db*) mice, activation of this pathway by PPAR*δ*-specific agonists is limited to the muscles. Administration of PPAR*β*/*δ* agonist ameliorates both muscle and liver insulin resistance in *db/db* mice by lowering the hepatic glucose output, increasing glucose disposal, and inhibiting fatty acid release from the adipose tissue. Surprisingly however, the expression arrays only detected induction of carnitine palmitoyltransferase 1 (*Cpt1*), a key gene in fatty acid *β*-oxidation, in the muscles, whereas the pathways responsible for the increased glucose disposal appeared to be hepatic fatty acid synthesis and pentose phosphate shunt that generates NADPH to provide reducing power for lipid synthesis [104]. Consistent with previous reports [105], PPAR*β*/*δ* agonist treatment did lead to increased *β*-oxidation rates in the muscles, suggesting that PPAR*β*/*δ* promote insulin sensitivity by consuming glucose through induction of hepatic fat-production in combination with a counterbalancing fat burning in muscle [104]. A recent study comparing hepatic gene regulation between wild type and PPAR*β*/*δ* -or PPAR*α* knockout mice showed that while PPAR*α* expression is highly upregulated during fasting, *P* *p* *a* *r* *β*/*δ* mRNA is downregulated. In accordance with this finding, the differences in gene expression between the wild type and PPAR*β*/*δ* knockout mice were more pronounced in the fed state but surprisingly a relatively large subset of genes were downregulated in a PPAR*β*/*δ*-dependent manner during the fast. Interestingly, there is only limited overlap between the hepatic genes regulated in a PPAR*α* or PPAR*β*/*δ*-dependent manner in the fed state, while a large proportion of the target genes appear to be regulated by both subtypes in the fasted state. It is evident that some of the differential effects on liver gene expression in the two knockout mouse models may be due to indirect effects imposed by other tissues; however, in agreement with the general perception of PPAR*β*/*δ* being an inhibitor of inflammation, pathway analyses showed that several gene sets involved in these processes were enriched in the knockout mice in the fed state. In the fasted state, the electron transport and oxidative phosphorylation pathways were decreased and in both metabolic states deletion of PPAR*β*/*δ* resulted in downregulation of genes involved in lipoprotein metabolism and carbohydrate metabolism, which included glycogen metabolism, glycolysis, gluconeogenesis, and the pentose phosphate pathway [106]. In agreement with the conclusion from the *db/db* mouse study indicating that PPAR*β*/*δ* is an important regulator of glucose disposal and utilization [104], these changes in gene expression resulted in significantly increased fasting plasma glucose levels in the PPAR*β*/*δ* knockout mice [106]. Besides studies of the PPAR*α* knockout mice [106, 107], other approaches to determine the PPAR*α* transcriptome includes overexpression studies in fibroblasts [16], exposure of hepatoma cell lines to synthetic PPAR*α* agonist [108], and *in vivo* examinations of the alterations in the gene expression pattern of mouse and monkey liver [109, 110] and mouse intestine [111] in response to agonist treatment. These studies unanimously report that PPAR*α* is the major inducer of *β*-oxidation in these tissues. In addition, it has also been reported that PPAR*α* function to repress amino acid catabolism [112].

The PPAR*γ* transcriptome of the adipogenic 3T3-L1 cell line has been characterized in several expression array studies because of the high endogenous expression level of PPAR*γ* and the observation that white adipose tissue is essential for the insulin sensitizing effects of the TZD PPAR*γ* agonists [113, 114]. Studies using these agonist to drive the differentiation of preadipocytes show that the TZDs are potent activators of adipogenesis and induce or enhance the expression of adipocyte specific markers and genes involved in lipid storage and transport, but also lipid hydrolysis and oxidation [115, 116]. Interestingly however, when mature adipocytes are exposed to TZDs for a few hours up to a couple of days, it leads to decreased expression of both PPAR*γ* itself and of lipid storage and adipokine genes while fatty acid activation and degradation is induced [116, 117]. The global effects of TZD treatment on gene expression has also been investigated in macrophages [118], colon cancer cells [119], aorta [120], and dendritic cells [121] with regulation of genes involved in lipid metabolism and inflammation being a consistent finding in these studies. 

Other approaches to annotate the PPAR*γ* transcriptome include analysis of NIH-3T3 fibroblasts transduced with retrovirus encoding PPAR*γ*2 [16, 122] and adenoviral overexpression of PPAR*γ*1 in the liver of PPAR*α* knockout mice for 2–6 days [20]. The latter resulted in hepatic steatosis, thus underscoring the lipogenic potential of PPAR*γ*. In order to increase the chances of identifying genes targeted directly by PPAR*γ* as opposed to genes being differentially expressed as a consequence of secondary regulation, we made use of acute adenoviral expression of the PPARs in NIH-3T3 fibroblasts, which have very low levels of endogenous PPARs [11]. This system allows us to induce rapid expression of the PPARs and subsequently evaluate the immediate effects on target gene activity at the mRNA level within 8 h after transduction, whereby secondary effects (e.g., induction of endogenous PPARs) on gene expression were minimized. By combining this cellular system with expression array analysis, we found that ectopic PPAR*γ*2 expression in combination with TZD treatment acutely activated a large number of known and novel target genes in the NIH-3T3 cells. Both expression of genes involved in lipid anabolic and catabolic pathways were induced but the net outcome was lipid accumulation [46]. These results corroborate previous findings from our lab that when expressed at adipocyte levels, PPAR*γ*2 is a strong transactivator capable of activating most PPAR target genes, even if other subtypes expressed at the same level would be better activators [11].

### 3.2. ChIP-Chip and ChIP-Seq Studies

The generation of genome-wide profiles of PPAR, RXR, and cofactor occupancy using the ChIP-chip, ChIP-seq, and ChIP-PET technologies has significantly increased the understanding of PPAR-mediated transactivation. First, these studies have shown that most target genes have multiple PPAR binding sites not only in the proximal promoter region but also in introns and at distant positions up- and downstream of the gene. Notably, about 50% of all PPAR:RXR-binding sites are found in introns. This distributions of binding sites reflects that of most other nuclear receptors [123–125] and of the several other transcription factors [126–128]. While these studies provide invaluable help in determining the position of binding sites, they also complicate the picture of functional PPREs and underscore the weaknesses of traditional promoter and enhancer characterization, where often only the proximal promoter or genomic sequences immediately upstream of the transcription start site are cloned in front of a luciferase reporter. 

The first PPAR cistrome to be published was that of PPAR*γ* in differentiating and mature 3T3-L1 adipocytes as mapped by ChIP-seq [129] and ChIP-chip [130]. Subsequently, the PPAR*γ* cistrome in 3T3-L1 cells has also been mapped by others using ChIP-chip [131], ChIP-seq [132], and ChIP-PET [133]. The mappings revealed between 2730 and 7000 genomic PPAR*γ* binding sites, depending on the method and false discovery rate employed. Notably, all genes encoding proteins involved in fatty acid handling and storage as well as lipolysis were found to have adjacent PPAR*γ*:RXR binding sites, but surprisingly this was also true for most genes encoding enzymes involved in steroid metabolism [133], glycolysis, the pentose phosphate pathway, and the TCA cycle [129, 131]. Interestingly, a significant overlap between PPAR*γ*:RXR binding sites and binding sites of CCAT/enhancer binding protein (C/EBP) *α* and -*β* was found [129, 130] indicating that the cooperativity between PPAR*γ* and members of the C/EBP family in the regulation of adipocyte gene expression (reviewed previously [134]) takes place on a much larger scale than previously anticipated. 

A recent study profiling the PPAR*γ* cistrome in primary mouse macrophages found that there was only very limited overlap between the genomic sites bound by the receptor in this cell type and in adipocytes. Interestingly, the transcription factor PU.1, which is involved in monocyte development and not expressed in adipocytes, was enriched at the macrophage specific sites. This study thus addresses the intriguing question of how cell type-specific PPAR*γ* transactivation is achieved at the level of chromatin binding and suggests that tissue-specific factors may play a role in facilitating PPAR*γ* binding to the tissue selective binding sites [132].

So far, genome-wide cistromes are not available for other PPAR subtypes, but recently PPAR*α* binding sites in a human hepatoma cell line was mapped by ChIP-chip using an array covering promoter regions from 7.5 kb upstream to 2.5 kb downstream of the transcription start site. This study showed increased binding of PPAR*α* to 4220 genomic regions in response to agonist treatment [135]. The group of genes assigned to these binding sites that were upregulated as determined by microarray analysis, clustered as being involved in sterol and lipid biosynthetic pathways, which is surprising given the general perception of PPAR*α* as an inducer of lipid degradation. The downregulated genes were involved in innate and humoral immune response, which is consistent with the well-described anti-inflammatory activity of PPAR*α* (Reviewed previously [5]).

## 4. Summary and Perspectives

As described in this paper, several molecular mechanisms conferring subtype-specificity to the PPARs or leading to preferential activation of a specific PPAR subtype in a certain cellular context have been elucidated. (1) The PPAR subtypes bind to the individual genomic PPREs with differential affinity. (2) The PPARs are activated by different ligands. (3) The PPAR subtypes display differential affinity towards various cofactors. (4) PPAR transcriptional activity is modulated by subtype-specific posttranslational modifications. (5) The PPAR A/B-domains potentiate subtype-specific activation of target genes while restricting nonselective target gene activation.

Most likely, PPAR subtype-specificity is maintained through the concerted effects of the regulatory mechanisms exerted by the individual PPAR domains or communicating PPAR and RXR domains. However, the current knowledge strongly suggests that the relative importance of these contributions is differential and that especially the A/B-domains are important mediators of PPAR subtype-specificity. Future studies should aim at pinpointing the exact sections of the A/B-and E-domains, and potentially the D-domain, that are involved in maintaining PPAR subtype-specificity and to fully elucidate the molecular mechanisms underlying this activity.

## Figures and Tables

**Table 1:** Cofactors regulating PPAR activity.

Coactivator	Enzymatic activity	Interaction	Reference
Bifunctional enzyme (BFE)	Dehydrogenase	*α* (A/B)	[57]
BRG1-associated factor 60c (BAF60c)	None	*γ* (A/B)	[58]
Coactivator-associated arginine methyltransferase 1 (CARM1)	HMT	SRC-1-3	[59]
Constitutive coactivator of PPAR*γ* (CCPG)	None	*γ* (D)	[60]
CREBP- binding protein (CBP)	HAT	*α*,*γ* (A/B, LBD, SRC)	[61]
Hydrogen peroxide-inducible clone 5 protein (Hic5)	None	*γ*	[62]
LIM domain-only protein (LMO4)	None	*γ* (D, LBD)	[63]
Lipin 1	None	*α*, PGC-1	[64]
Mediator subunit 1 (MED1)	None	*α*, *β*/*δ*,*γ* (LBD)	[65]
Mediator subunit 14 (MED14)	None	*γ* (A/B)	[66]
Multiple Endocrine Neoplasia type 1 (MEN1)	None	*γ* (LBD)	[67]
Multiprotein bridging factor 1 (MBF-1)	None	*γ* (D, LBD)	[68]
Murine double minute 2 (MDM2)	Ubiquitin ligase	*α*, *β*/*δ* (A/B)	[69]
p300	HAT	*α*,*γ* (A/B, LBD, SRC)	[61]
Poly ADP-ribose polymerase 2 (PARP-2)	ADP-ribose polymerase	*γ*	[70]
Positive transcription elongation factor b complex (P-TEFb)	Kinase	*γ*	[71]
PPAR*α*-interacting complex 285 (PRIC285)	DNA helicase	*α*, *β*/*δ*, *γ* (DBD)	[72, 73]
PPAR*α*-interacting complex 320 (PRIC320)	DNA helicase	*α*	[74]
PPAR-interacting protein (PRIP)	None	*α*,*γ* (LBD)	[75]
PPAR*γ* coactivator 1*α* (PGC-1*α*)	None	*α*,*γ* (DBD)	[76]
PPAR*γ* coactivator 2 (PGC-2)	None	*γ* (A/B)	[15]
PR domain containing 16 (PRDM16)	None	*α*,*γ*	[77]
PRIP-interacting protein with methyltransferase domain (PIMT)	HMT	PRIP, CBP, MED1	[78]
Protein arginine N-methyltransferase 2 (PRMT2)	HMT?	*γ*	[79]
Steroid receptor coactivator-1 (SRC-1)	HAT	*α*, *β*/*δ*, *γ* (LBD)	[80]
Steroid receptor coactivator-2 (SRC-2)	HAT	*α*,*γ* (LBD)	[81]
Steroid receptor coactivator-3 (SRC-3)	HAT	*α*, *β*/*δ*,*γ* (LBD)	[81]
Tat interactive protein (Tip60)	HAT	*γ* (A/B)	[82]
Thyroid hormone receptor interacting protein 3 (TRIP3)	None	*γ* (LBD)	[83]

Corepressor	Enzyme activity	Interaction	Reference

Histone deacetylase 1 (HDAC1)	HDAC	NCoR, SMRT	[84]
Histone deacetylase 3 (HDAC3)	HDAC	NCoR, SMRT	[85, 86]
Insulin-like growth factor-binding protein-3 (IGFBP-3)	None	*γ*	[87]
Nuclear receptor corepressor 1 (NCoR)	None	*α*, *β*/*δ*, *γ*	[88]
Receptor*-*interacting protein 140 (RIP140)	None	*α*,*γ* (LBD)	[89]
Scaffold attachment factor B1 (SAFB1)	None	*α*, *β*/*δ*, *γ*	[90]
Silencing mediator of retinoid and thyroid receptors (SMRT)	None	*α*, *β*/*δ*, *γ*	[88]
Silent mating type information regulation 2 homolog 1 (SIRT1)	HDAC	NCoR, PGC-1	[91]
Tribbles homolog 3 (TRB3)	None	*γ* (A/B)	[92]

Histone methyltransferase (HMT), Histone acetyltransferase (HAT), and Histone deactylase (HDAC).
